# Editorial: Nutrition management puzzle in IBD

**DOI:** 10.3389/fmed.2025.1770981

**Published:** 2026-01-15

**Authors:** Sara Jarmakiewicz-Czaja, Rafał Filip

**Affiliations:** 1 Faculty of Health Sciences and Psychology, Collegium Medicum, University of Rzeszów, Rzeszów, Poland; 2 Department of Gastroenterology with IBD Unit, St. Jadwiga Queen Hospital No. 2 Affiliated to the Medical College, University of Rzeszow, Rzeszów, Poland; 3 Department of Internal Medicine, Medical College, University of Rzeszow, Rzeszów, Poland

**Keywords:** body composition, diet, inflammatory bowel diseases (IBD), malnutrition, vitamin D

## Introduction

Inflammatory bowel disease (IBD), including Crohn's disease (CD) and ulcerative colitis (UC), belongs to a group of gastrointestinal diseases. It progresses with periods of active and remission phase. The etiology of IBD is not fully understood, but it is often complex and involves several different factors, such as genetic, immunological, environmental, and gut microbiota dysbiosis (1, 2). Currently, Western countries are characterized by a rapid incidence of IBD, ranging from 12 to 26 per 100,000 people (3). Due to the symptoms of the disease, in addition to pharmacological and surgical treatment, proper nutrition is a very important aspect that supports basic therapy.

During remission, a healthy diet that avoids individual nutritional risk factors is recommended for IBD patients, while according to the ESPEN guidelines, there is no diet that accelerates remission in IBD (4). Wei et al. analyzed the effect of certain diets on specific parameters in patients with IBD, including C-reactive protein (CRP) levels, albumin (ALB) levels, and disease activity assessed endoscopically using the Mayo Endoscopic Score (MES). The authors observed that the combination of a low-FODMAP diet with enteral nutrition significantly reduced CRP levels compared to a standard diet, a low-residue diet, and a low-FODMAP diet without modification. Similarly, these two diets proved to be the most effective in increasing albumin levels. In terms of endoscopic remission, the researchers observed the best results after following a diet based on IgG testing (IgG-ED). There is no universal diet for IBD, and different clinical goals require different nutritional approaches.

One of the most common deficiencies in patients with IBD is vitamin D deficiency. This can have serious clinical consequences in the form of impaired immune function, osteopenia, osteoporosis, and others. In their study, Kojecký et al. compared classic oral vitamin D supplementation with an aerosol form for administration through the oral mucosa. The researchers observed that in order to maintain stable serum levels above 75 nmol/L, the required dose of the oral absorbed form is approximately 1,000 IU/day, while oral supplementation is approximately 2,000 IU/day. The possibility of achieving adequate 25OHD concentrations at a lower dose and without absorption problems is of practical importance. That is why it is so important to recommend the appropriate dose of vitamin D to patients.

Patients with IBD may develop malnutrition or cachexia due to various factors, including inflammation, dietary restrictions, improper diet, medication, rapid weight loss, etc. (5, 6). Desalegn et al. indicate that more than one-third of patients can be classified as at risk of malnutrition or already malnourished, which can increase the burden on healthcare. They observed that lower monthly income and a high Malnutrition Universal Screening Tool (MUST) risk score can be independently associated with a higher risk of IBD activity. Dietary interventions, nutrition education, socioeconomic support, and monitoring of the nutritional status of patients with IBD are key elements of the treatment strategy.

Huang et al. observed in their study that there is a relationship between dynamic body composition parameters, e.g., muscle tissue, and the risk of surgical intervention. They also indicate that the introduction of appropriate dietary and physical interventions to reduce sarcopenia could potentially reduce surgical risk.

Early implementation of enteral nutrition when necessary, anti-inflammatory diets with proven efficacy, adequate protein intake, or personalized nutritional interventions can be a preventive measure against progression, not just a way to alleviate symptoms (7, 8).

## Conclusions

Properly selected nutrition can have a real and measurable impact on the course of IBD, from laboratory results, nutritional status, to the risk of complications or disease recurrence. It is worth ensuring comprehensive, individualized nutritional care as an integral part of therapy. Properly selected nutrition can bring real benefits to patients with IBD.
